# Childhood Systemic Lupus Erythematosus; a Rare Multisystem Disorder: Case Report of a 3-year-old Girl with Oral Involvement as a Primary Sign

**DOI:** 10.30476/DENTJODS.2019.77699.0

**Published:** 2020-12

**Authors:** Azadeh Horri, Masume Danesh, Maryam Sadat Hashemipour

**Affiliations:** 1 Oral and Dental Research Center, School of Dentistry, Kerman University of Medical Sciences, Kerman, Iran; 2 Dept. of Pediatrics, Kerman University of Medical Sciences, Kerman, Iran; 3 Dept. of Oral Health, Oral and Dental Research Center, School of Dentistry, Kerman University of Medical Sciences, Kerman, Iran

**Keywords:** Systemic Lupus Erythematosus (SLE), Herpetic gingivostomatitis, Autoimmune disorder

## Abstract

Childhood-onset systemic lupus erythematosus (cSLE) is a severe, chronic, multi-organ, and systemic autoimmune disorder characterized by inflammatory and autoimmune reaction in several organs. The occurrence of systemic lupus erythematosus (SLE) in children is very rare. About 20% of all SLE cases are diagnosed during the first two decades of life and the disease is extremely rare before age of 5 years. In this case report, we present a 3-year-old girl presented with SLE symptoms similar to primary herpetic gingivostomatitis. Early diagnosis lead to proper treatment of the disease and it is important to decrease oral complications in children. Diagnosis could be improved by introduce new cases to provide valuable information for dentists based on diagnostic criteria, therapeutic steps and complication of treatment of SLE in Children. Therefore, it could be concluded that dentists involved in pediatric dentistry should consider and work out on the clinical signs of SLE in children with history of oral herpes virus infection.

## Introduction

Childhood-onset systemic lupus erythematosus (cSLE) is a severe, chronic, multi-organ, systemic autoimmune disorder presented by inflammatory and autoimmune reaction in multiple organs [ 1 - 2 ]. Although the cSLE have the same pathophysiology compared to adult type SLE, but the initial clinical presentation of cSLE is observed more sever [ 3 - 8 ]. In addition, the abnormal appearance common in this age group is frequently responsible for major diagnostic delay [ 5 ]. Considering the most report series, the oral lesions are minor [ 4 - 10 ], therefore, they usually do not come to dental pediatric centers. The incidence of cSLE varies from 0.3 to 0.9 per 100,000 children-years [ 11 - 12 ]. It has been demonstrated that both renal and central nervous system (CNS) organs tend to be more involved in pediatric patients than in adults [ 13 - 14 ].

Only 20% of all patients with SLE are diagnosed during the first two decades of life and the disease is extremely rare in those below 5 years of age [ 1 , 15 ]. The diagnosis and treatment of patients with cSLE is based on the European evidence-based recommendations for diagnosis and treatment of cSLE [ 16 ]. Although several studies have reported the clinical and laboratory characteristics of patients with cSLE [ 1 , 3 , 17 ], based on literature review, there is no presentation of the disease in younger children (below 5 years of age). In this paper, we report a cSLE case of 3- year-' old girl, referred to Pediatric Department, Dental School, Medical University of Kerman, Iran, to provide a valuable information for dentists to improve diagnostic criteria, therapeutic steps in children with oral complications. 

## Case Report

A 3-year-old girl was referred to Pediatric Dentistry Department with severe white (keratotic) lesions on palate and buccal mucosa in oral cavity and lips along with odynophagia and inability to eat for three months. She had general gingival involvement and received analgesics and antibiotics including acetaminophen and Cefixime without any recovery. She had a history of malaise, fever, and fatigue at the onset of the disease along with weight loss (4 kg during 3 months). After one month, were observed that oral and facial lesions were gradually progressed.

 On physical examination, there was no lesion in the mucosa of eye and nose while a pale lesion on the face especially around the cheek and nose was observed. There were some cutaneous lesions on her scalp but no lesions on arms or legs. She had no history of primary gingivostomatitis. A positive family history (her grandfather and uncle) of rheumatoid arthritis was reported. The patient was the third and the last child of the family, being 15years younger than her siblings. There was no history of congenital disorders. Consultation with the Department of Diagnosis of Oral Diseases was carried out and according to the age of the child and acute oral symptoms, the primary diagnosis of the disease was established as primary herpetic gingivostomatitis according to epidemiology, clinical sign, and history. Based on response to the treatment regimen, other differential diagnoses including aphthous lesion, vesiculo bullous lesions, and Hand-Foot-Mouth disease were ruled out.

**Figure 1:**
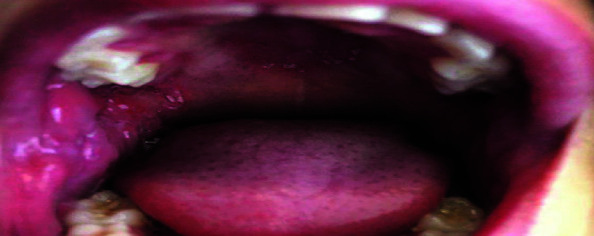
Oral lesions, ulcer in hard palate and buccal mucosa

The first impressions of patient were considered primary herpetic gingivostomatitis and the girl was treated with Nystatin suspension, Diphenhydramine, and Magnesium aluminum. The patient was followed up by supportive care and after 10 days, the oral aphthous lesions and the malar rash were exacerbated (Figure 1). Concerning the worsening of the lesions, further evaluations were scheduled. Subsequently by assessing the complete blood cell count and differential count (CBC &amp;diff), biopsy of facial lesion (patient did not cooperate for oral lesion biopsy), and consultation with a rheumatologist, the SLE was diagnosed. 

Blood investigations revealed a hemoglobin concentration of 15.4 g/dL, and white blood cell count of 13.2× 109/L and platelet count of 486×109/L. The lymphocyte percentage was 58% compared to 35% neutrophil. Her erythrocyte sedimentation rate (ESR) was 31 mm at the first hour, and C-reactive protein (CRP) was positive. Her serum creatinine level was 0.52 mg/dL, and blood urea nitrogen (BUN) was 14 mg/dL. To rule out SLE and other collagen vascular diseases, the serologic tests showed positive antinuclear antibody (ANA), negative Anti-dsDNA and positive cytoplasmic antineutrophil cytoplasmic antibodies (C-ANCA). The serum level of C3-C4 complement fractions were reported normal (Table 1). Skin biopsy from the malar rash revealed non-specific chronic inflammation (dermatitis) with mononuclear cells without viral inclusion bodies.

**Table 1:** Laboratory findings of patient

Test	Result	Normal Value
W.B.C	13000	4000-11000
R.B.C	5.02	4.3-5.2×106
Hemoglobin	13.5	12-16
Hematocrit	41	47-52
M.C.V	81.7	78-98
M.C.H	26.9	21-26
M.C.H.C	32.9	32-36
Platelet	386000	150000-400000
Creatinine	0.52	0.5-1.1
AST	37	15-45
ALT	20	15-45
ALKP	355	180-1200
ZN	71	70-120
ESR	31	0-20
CH50	90	101-300
Anti-dsDNA	9	+/-
C3	1.58	83-177 mg/dl
C4	0.35	15-45 mg/dl
ANA	3.5	1.2
P-ANCA	1.2	-
C-ANCA	10.6	-
G6PD	NORMAL	-
ANA-Screen	1.1	-
CRP Quantitative	9	0/8-4

ALKP: alkaline phosphatase; ALT: Alanine Aminotransferase; ANA: antinuclear antibodies; AST: aspartate; Aminotransferase; CRP: C-reactive protein ESR: erythrocyte sedimentation rate; G6PD: Glucose-6-phosphate dehydrogenase; MCH: mean corpuscular hemoglobin; MCHC: mean corpuscular hemoglobin concentration; MCV: mean corpuscular volume; RBC: red blood cell; WBC: white blood cell

Malar rash for face skin lesion was observed. In performed biopsy, the IgG and shaggy basement membrane zone was positive while IgM and C3 were negative. There was epidermal atrophy, mononuclear cell infiltration and orthokeratosis but there was no blister for mation. The pathologist differential diagnosis included lupus erythematosus and pemphigus vulgaris. 

**Figure 2:**
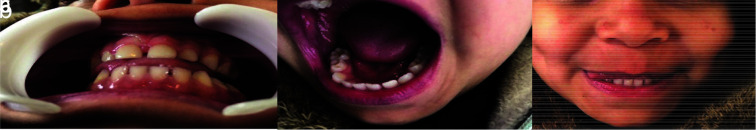
**a:** Gingival lesion was recovered, **b:** The oral aphthous lesions of the patient after 10 days of conservative therapy, **c:** Pallor malar rash

After consultation with a neurologist and a nephrologist, it was confirmed that CNS and the renal system were not involved considering the normal urinalysis. Based on the clinical findings and the laboratory results (6 out of 11 criteria), the diagnosis of cSLE was confirmed regarding the American College of rheumatology (ACR) (Table 2) revised criteria for diagnosis of SLE [ 18 ]. The positive criteria included malar rash, oral ulcers, discoid rash (scalp lesions), photosensitivity in malar rash, antinuclear antibody, immunologic disorder (positive ANA, c-ANCA, and CRP). A rheumatologist was consulted and oral prednisolone (5 mg, twice daily) and oral hydroxychloroquine (5mg/kg once daily) was administered. The patient was followed up and after 10 days of therapy, the oral aphthous lesions were recovered and the malar rash disappeared (Figure 2). The patient was able to eat and drink and her weight increased after a month. In 6-month follow-up visit, there was no relapse and no lesion was detected. There was no renal and CNS involvement. The patient was recommended to continue the therapeutic regimen until 6 years of age. Relevant written consents have been obtained before publishing the case report.

**Table 2:** The revised criteria for classification of systemic lupus erythematosus (1982)

1. Malar rash: Fixed erythema, flat or raised, over the malar eminences, tending to spare the nasolabial folds
2. Discoid rash: Erythematous raised patches with adherent keratotic scaling and follicular plugging; atrophic scarring may occur in older lesions:
3. Photosensitivity: Skin rash as a result of unusual reaction to sunlight, by patient history or physician observation
4. Oral ulcers: Oral or nasopharyngeal ulceration, usually painless, observed by physician
5. Arthritis: Nonerosive arthritis involving 2 or more peripheral joints, characterized by tenderness, swelling, or effusion
6. Serositis: a) Pleuritis--convincing history of pleuritic pain or rubbing heard by a physician or evidence of pleural effusion OR b) Pericarditis--documented by ECG or rub or evidence of pericardial effusion
7. Renal disorder: a) Persistent proteinuria greater than 0.5 grams per day or greater than 3+ if quantitation not performed OR b) Cellular casts--may be red cell, hemoglobin, granular, tubular, or mixed
8. Neurologic disorder: a) Seizures--in the absence of offending drugs or known metabolic derangements; e.g., uremia, ketoacidosis, or electrolyte im-balance OR b) Psychosis--in the absence of offending drugs or known metabolic derangements, e.g., uremia, ketoacidosis, or electrolyte imbalance
9. Hematologic disorder: a) Hemolytic anemia--with reticulocytosis OR b) Leukopenia--less than 4,000/mm3total on 2 or more occasions OR c) Lyphopenia--less than 1,500/mm3on 2 or more occasions OR d) Thrombocytopenia--less than 100,000/mm3in the absence of offending drugs
10. Immunologic disorder: a) Positive LE cell preparation OR b) Anti-DNA: antibody to native DNA in abnormal titer OR c) Anti-Sm: presence of antibody to Sm nuclear antigen OR d) False positive serologic test for syphilis known to be positive for at least 6 months and confirmed by Treponema pallidum immobilization or fluorescent treponemal antibody absorption test
11. Antinuclear antibody: An abnormal titer of antinuclear antibody by immunofluorescence or an equivalent assay at any point in time and in the absence of drugs known to be associated with "drug-induced lupus" syndrome

## Discussion

Infantile SLE is an extremely rare and to the best of our knowledge, only 13 cases have been reported in the world [ 19 - 20 ] (Table 3). The clinical course of the cSLE is progressive and associated with more severe symptoms and more progressive course with permanent sequellae [ 1 , 3 , 14 ]. Of the 14 patients reported in the literature, 5 died and 6 developed end-stage renal failure and complication of the CNS. It has been previously demonstrated that the prognosis of infantile SLE with high-grade glomerulonephritis is poor [ 19 , 21 - 22 ]. It is interesting that our patient had a benign course and did not have any evidence of lupus nephritis or involvement of the CNS. Evidence for a genetic predisposition to SLE in humans is based on the concordance rate (23%-57%) saw in indistinguishable twins and on the relative high frequency of familial cases (8%-12%). Candidate genes or loci for SLE liability have been located on the long-arm of chromosome 1 [ 23 ]. Our case had a family history of rheumatoid arthritis for her grandfather and uncle. Previously, Zulian *et al*. [ 20 ] demonstrated that infantile SLE had a more progressive course and was associated with more destructive symptoms than adult SLE. They also reported anemia and thrombocytopenia as frequent findings in infantile SLE, whereas eucopenlia was rare. Conversely, leukocytosis has been reported to be more common in infantile SLE [ 3 , 20 ]. In our case, there was no anemia and thrombocytopenia but the patient had leukocytosis in which the lymphocyte was more dominant. These hematological abnormalities, together with positive ANA and decreased C3-C4 complement fractions, should address the diagnosis of SLE in young children with unexplained fever, irritability, and rash. However, since positive ANA and hypocomplementemia may be because of concomitant infections, these findings have low specificity as diagnostic tools, especially, in this age group, where SLE has a very low prevalence [ 20 ]. Currently, intravenous cyclophosphamide (IVCY) therapy is considered the standard treatment for both children and adults with severe lupus nephritis [ 16 ]. However, in our patient, as there was no evidence of lupus nephritis, we did not administer the IVCY. The efficacy of IVCY in treatment of infantile SLE is yet to be identified. There are only two previous reports of successful IVCY treatment, in infantile SLE with lupus-nephritis [ 19 , 22 ]. 

**Table 3:** Previous reports of cSLE

Reference No	[24]	[25]	[26]	[27]	[28]	[29]	[16]
Patient No	1	2	3	4	6	7	8
Age at onset (months)	11	2.5	5	9	3	3	8
Sex	F	F	F	F	F	M	M
Race	African-American	Caucasian	Caucasian	Caucasian	Caucasian	Caucasian	Caucasian
Signs and symptoms at disease onset	Fever, Rash Alopecia Oral ulcers	Fever, Serositis Edema	Rash Hypertonia, RASH	Fever	Fever, Diarrhea	Irritability Purpuric Rash Oral ulcers	Irritability Purpuric Rash Oral ulcers
First diagnosis	Multiforme erythema	SLE	DIC	DIC	SLE	SLE	SLE

DIC=disseminated intravascular coagulation ; SLE= systemic lupus erythematosus

As recommended by the European evidence-based recommendations for diagnosis and treatment of cSLE, corticosteroids remain the mainstay of the treatment of infantile and cSLE. All the previously reported cases received high dosages of prednisolone with different responses [ 3 , 19 - 22 ]. In the current report, we observed significant and abrupt response of the symptoms to the initial dose of prednisolone. However, in previously reported cases, the response to the corticosteroids were limited and thus adding other agents were required [ 3 , 19 - 20 ]. In brief, the rare case of infantile SLE can be treated successfully treated with oral prednisolone. Timely diagnosis and treatment is the key step in treatment of cSLE. The diagnosis in this age group should be based on the clinical suspicion. 

## Conclusion

Since cSLE is extremely rare in individuals younger than 5 years of age, and concerning the oral symptoms of SLE in children, dentists' awareness of these symptoms leads to earlier diagnosis and therefore better treatment. 
